# Diversity of selected toll-like receptor genes in cheetahs (*Acinonyx jubatus*) and African leopards (*Panthera pardus pardus*)

**DOI:** 10.1038/s41598-024-54076-y

**Published:** 2024-02-14

**Authors:** René Meißner, Prudent Mokgokong, Chantelle Pretorius, Sven Winter, Kim Labuschagne, Antoinette Kotze, Stefan Prost, Petr Horin, Desire Dalton, Pamela A. Burger

**Affiliations:** 1 Research Institute of Wildlife Ecology, University of Veterinary Medicine, Savoyenstraße 1, 1160 Vienna, Austria; 2 South African National Biodiversity Institute, National Zoological Garden, 232 Boom Street, Pretoria, 0002 South Africa; 3 WWF South African, Bridge House, Boundary Terraces, Mariendahl Ave, Newlands, 7725 Capetown South Africa; 4 University of the Free State, Bloemfontein Campus, Bloemfontein, 9300 South Africa; 5 University of Oulu, Pentti Kaiteran Katu 1, 90570 Oulu, Finland; 6 Department of Animal Genetics, University of Veterinary Sciences, Brno, Czech Republic; 7 Central European Institute of Technology, University of Veterinary Sciences Brno (CEITEC Vetuni), Brno, Czech Republic; 8 School of Health and Life Science, Teesside University, Middlesbrough, Tees Valley TS1 3BX UK

**Keywords:** Evolutionary biology, Conservation biology

## Abstract

The anthropogenic impact on wildlife is ever increasing. With shrinking habitats, wild populations are being pushed to co-exist in proximity to humans leading to an increased threat of infectious diseases. Therefore, understanding the immune system of a species is key to assess its resilience in a changing environment. The innate immune system (IIS) is the body’s first line of defense against pathogens. High variability in IIS genes, like toll-like receptor (TLR) genes, appears to be associated with resistance to infectious diseases. However, few studies have investigated diversity in TLR genes in vulnerable species for conservation. Large predators are threatened globally including leopards and cheetahs, both listed as 'vulnerable' by IUCN. To examine IIS diversity in these sympatric species, we used next-generation-sequencing to compare selected TLR genes in African leopards and cheetahs. Despite differences, both species show some TLR haplotype similarity. Historic cheetahs from all subspecies exhibit greater genetic diversity than modern Southern African cheetahs. The diversity in investigated TLR genes is lower in modern Southern African cheetahs than in African leopards. Compared to historic cheetah data and other subspecies, a more recent population decline might explain the observed genetic impoverishment of TLR genes in modern Southern African cheetahs. However, this may not yet impact the health of this cheetah subspecies.

## Introduction

The innate immune system (IIS) is the genetically predetermined response to foreign substances in most multicellular organisms^1^. As the body’s first line of defense, its ability to recognize pathogens is crucial to initiate countermeasures and induce defensive reactions^2^. Nevertheless, foreign substances are also detected by the adaptive immune system (AIS), e.g., by T cell antigen receptors, but this defense mechanism is more specific and only found in jawed vertebrates^3^. Among IIS-specific receptors that recognize conserved patterns on microorganisms are the toll-like receptors (TLRs)^4^. TLRs are type I transmembrane proteins either located on the cell surface or within the cell compartment and are grouped by their protein sequence similarity. Structurally, they consist of a leucine-repeat rich ectodomain (LRR), which detects pathogen-associated molecular patterns (PAMPs), a transmembrane domain, and a cytoplasmic domain Toll-IL1 receptor (TIR) that initiates the intracellular signaling cascade^5^. In mammals, there are at least 13 members of the TLR protein family, each with a designated role in recognizing specific pathogens^6^. Most TLRs detect bacterial components with a varying specificity to different ligands, e.g., TLR1 and TLR6 respond to different lipopeptides of the bacterial lipoprotein^7^, while TLR4 in particular recognizes lipopolysaccharides in Gram-negative bacteria^4^. Most TLRs located within the cell compartment respond to foreign nucleotides, and thus can detect viruses and parthenogenic protozoans^8,9^. Therefore, TLRs are essential in signal amplification, induce protein trafficking pathways to trigger inflammatory responses and induct the adaptive immune system (AIS)^10^.

Generally, a species’ resilience against infectious diseases is linked to high genetic diversity^11,12^. Correspondingly, genes associated with the immune system are considered to be among the most polymorphic due to adaptive evolution^13–15^. In contrast to major parts of the AIS, standing genetic variations is the only source of diversity for the IIS^2^. It is assumed that the number of polymorphism in innate immunity receptors affects a species’ ability to adapt to future environmental changes^16,17^. Especially the variability in TLR genes seems to be connected to resistance against infectious diseases because increased variation enhances the potential for binding a larger variety of PAMPs^17,18^. Though important, only few studies have investigated diversity in TLR genes in non-model organisms and drawn conclusions for conservation^19–21^ while often lacking comparisons to a related species. Studies on felids are especially rare and either focus exclusively on the domestic cat (*Felis catus*)^22–24^ or are limited by their extent if other species are included^25^. However, a species’ immune fitness can only be drawn from the context, as a detached evaluation of genetic diversity does not easily allow meaningful conclusions^26^.

Throughout Africa, many essential niches, especially in the case of large carnivores, are occupied by big cats such as lions (*Panthera leo*), leopards (*Panthera pardus*), and cheetahs (*Acinonyx jubatus*), which serve important ecosystem functions^27,28^. Savannas, in particular, profit from those apex predators because the trophic impact exacted by carnivores strongly shapes and maintains an ecosystem’s balance^29–31^. In Sub-Saharan Africa, cheetahs and leopards inhabit similar open habitats and often share a sympatric distribution^32,33^. Their coexistence is a result of niche partitioning. While leopards are opportunistic hunters of larger prey, cheetahs are specialized in small fast-running antelopes and lagomorphs^34,35^. Due to increasing anthropogenic pressure they frequently occur near settlements, with leopards more prone to direct human contact^36,37^ while cheetahs rather interact with livestock^38,39^. Nevertheless, both species occasionally hunt stock animals^40–42^ and therefore, face more active persecution than, e.g., lions which rarely occur outside protected areas^43^. As a direct consequence of this ever-growing human-wildlife conflict and other factors such as habitat destruction, leopard and cheetah are listed as ‘vulnerable’ on the International Union for Conservation of Nature (IUCN) Red List of Threatened Species^44^. Still, the conservation status of different subspecies varies greatly and negatively correlates with increasing human impact^45,46^. Three of the nine leopard subspecies and two of the five classical cheetah subspecies are considered ‘critically endangered’^44,47,48^. With fewer than 50 individuals in the wild, the Asiatic cheetah (*A. j. venaticus*) is already facing extinction^49,50^.

Beyond these threats, however, proximity to humans poses a less apparent danger: infectious diseases. These pathogens, either transferred by vectors or directly by domestic animals like feral cats, further pressure wild populations^51,52^. Because of a similar solitary lifestyle, it can be assumed that infectious diseases might affect both species similarly. However, leopards and cheetahs are less similar on the genetic diversity level. Especially the African leopard is known to be among the most genetically diverse big cat species, with high heterozygosity and no apparent structure despite its decreasing populations^53^. On the contrary, cheetahs are classically portrayed as very homogeneous and overall genetically impoverished^54,55^ due to a proposed bottleneck event 10,000 years ago^56,57^. Nonetheless, recent studies observe more diversity than previously thought, e.g., for immune gene loci such as the major histocompatibility complex (MHC)^58,59^. Yet, cheetahs show strong geographical differentiation^47,60,61^ as well as low genome-wide diversity^61^. Here, we compare TLR2, TLR4, TLR6 and TLR8 of modern Southern African cheetahs (*A. j. jubatus*; n = 49) to sympatric occurring African leopards (*P. p. pardus*; n = 41) and incorporate historic cheetah samples and samples of different cheetah subspecies (*A. j. ssp.*: n = 15) into our study as a temporal-spatial reference. All four TLRs genes play a major role in the initial detection and defense against infectious diseases by primarily detecting bacterial (TLR2, TLR4, TLR6) and viral (TLR8) PAMPs. Therefore, their underlying genetic diversity can be an indication of a species resilience against emerging infectious threats.

## Results

Despite successful preliminary testing of all five primer sets of our target TLR exons using three modern cheetah samples, we excluded exon 1 of TLR4, which failed in subsequent amplifications in most samples. From our 61 modern Southern African cheetah samples (Supplementary Table 1) we discarded 12 samples that failed initial amplification. All results are based on exon data of TLR2, TLR4.2, TLR6, and TLR8, deposited in the Phaidra public repository (https://doi.org/10.34876/9KFD-2A38).

### Genetic diversity in TLR exons of African leopards and cheetahs

**Figure 1:**
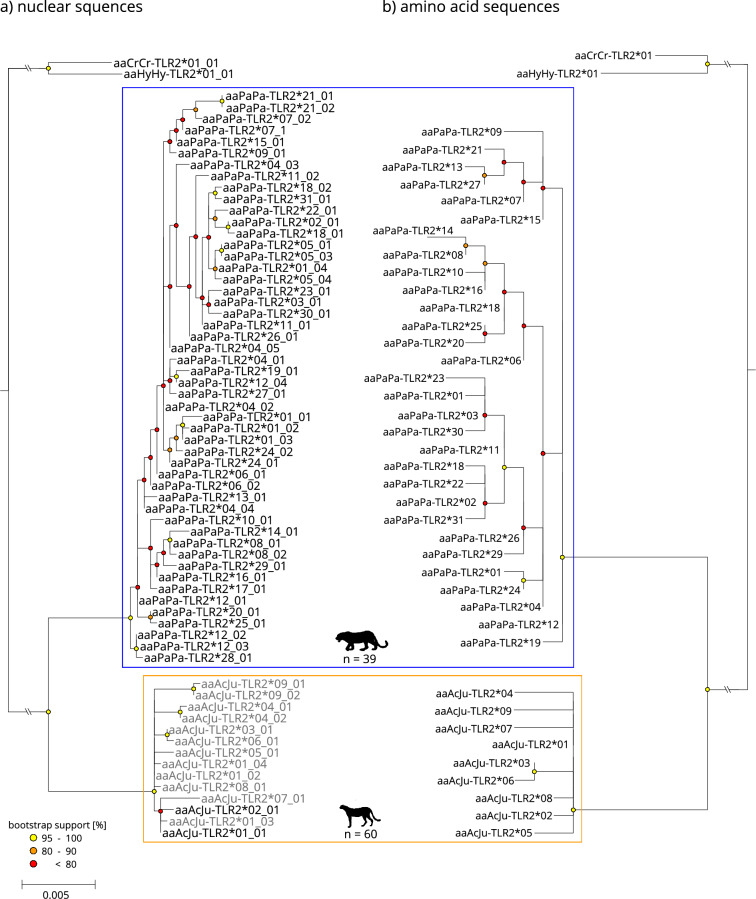
Maximum-likelihood phylogeny of the TLR2 exon sequences (**a**) and the resulting amino acid sequences (**b**) for leopard (blue framed) and cheetah (orange framed), faded tip labels indicate nucleotide alleles/resulting amino acid sequences only occurring in historic samples. The scale indicates the number of substitutions per side. Spotted hyena (*Crocuta Crocuta*, aaCrCr) and striped hyena (*Hyaena hyaena*, aaHyHy) were used as an outgroup.

**Figure 2:**
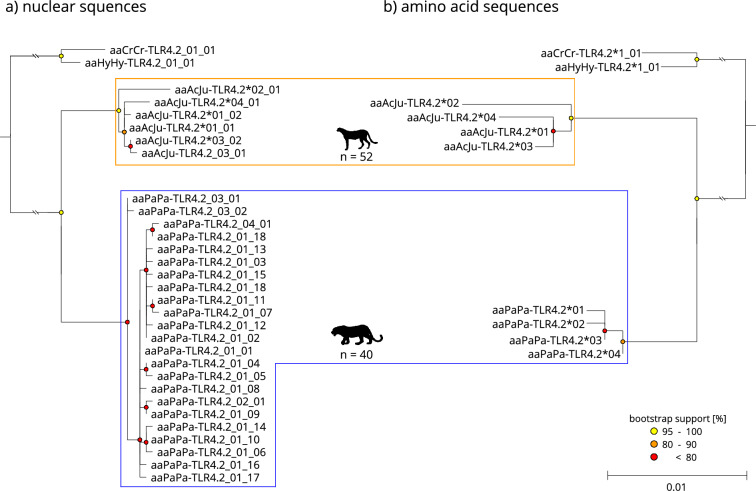
Maximum-likelihood phylogeny of the TLR4.2 exon sequences (**a**) and the resulting amino acid sequences (**b**) for leopard (blue framed) and cheetah (orange framed), faded tip labels indicate nucleotide alleles/resulting amino acid sequences only occurring in historic samples. The scale indicates the number of substitutions per side. Spotted hyena (*Crocuta Crocuta*, aaCrCr) and striped hyena (*Hyaena hyaena*, aaHyHy) were used as an outgroup.

**Figure 3:**
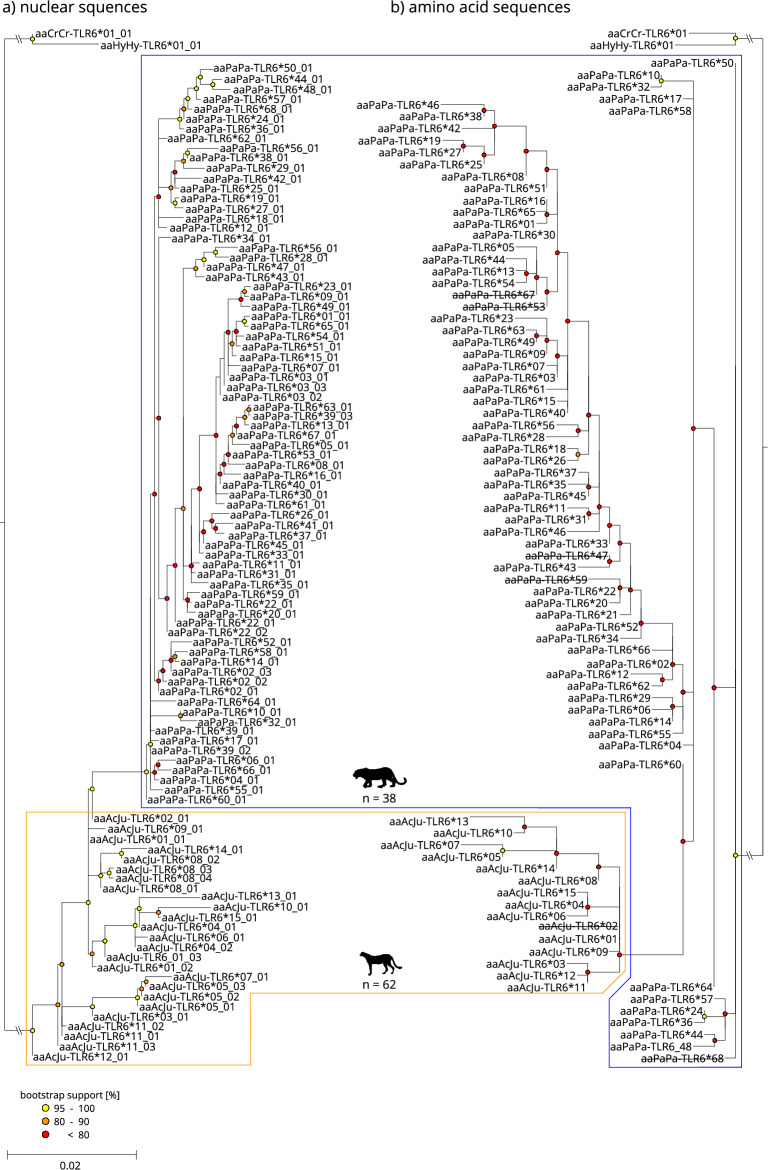
Maximum-likelihood phylogeny of the TLR6 exon sequences (**a**) and the resulting amino acid sequences (**b**) for leopard (blue framed) and cheetah (orange framed), faded tip labels indicate nucleotide alleles/resulting amino acid sequences only occurring in historic samples. Crossed-out tip labels indicate likely functionless amino acid sequences due to deletions resulting in preliminary stop codons. The scale indicates the number of substitutions per side. Spotted hyena (*Crocuta Crocuta*, aaCrCr) and striped hyena (*Hyaena hyaena*, aaHyHy) were used as an outgroup.

**Figure 4:**
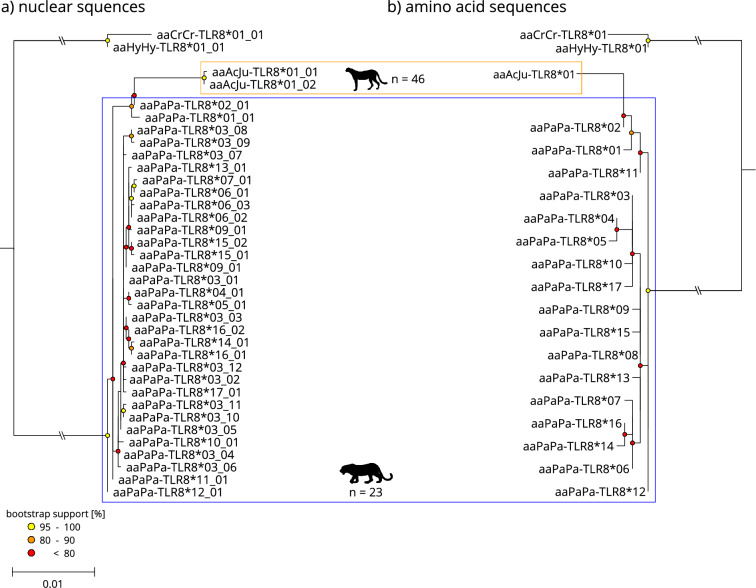
Maximum-likelihood phylogeny of the TLR8 exon sequences (**a**) and the resulting amino acid sequences (**b**) for leopard (blue framed) and cheetah (orange framed), faded tip labels indicate nucleotide alleles/resulting amino acid sequences only occurring in historic samples. The scale indicates the number of substitutions per side. Spotted hyena (*Crocuta Crocuta*, aaCrCr) and striped hyena (*Hyaena hyaena*, aaHyHy) were used as an outgroup.

All investigated TLR exons showed different haplotype diversity between African leopards and cheetahs varying in total nucleotide allele count, resulting amino acid sequences, phylogenetic structure and diversity (Table 1, Figs. 1, 2, 3, 4).

**Table 1:** TLR diversity comparison between African leopards, modern Southern African cheetahs, and historic cheetahs of different subspecies.

	n	N_alleles_	N_amino acid_	H_obs_	H_esp_	h_div_
TLR2						
Leopard_Afr_	39	50	31	100	98.3	0.9827
Cheetah_SA_	45	2	2	2.2	2.2	0.0222
Cheetah_his_	15	13	8	80	79.5	0.7806
TLR4-2						
Leopard_Afr_	40	23	4	55	86.2	0.8617
Cheetah_SA_	40	2	2	2.5	2.4	0.025
Cheetah_his_	12	5	3	50	65.6	0.6581
TLR6						
Leopard_Afr_	38	75	68	100	100	0.9996
Cheetah_SA_	48	5	3	58.3	55.6	0.4974
Cheetah_his_	14	22	12	92.9	95.8	0.9606
TLR8						
Leopard_Afr_	23	32	17	78.3	97.8	0.9778
Cheetah_SA_	37	1	1	0	0	0
Cheetah_his_	8	2	1	12.5	45.8	0.5497

African leopards [Leopard_Afr_], modern Southern African cheetahs [Cheetah_SA_], historic cheetahs of different subspecies [Cheetah_his_], number of samples used [n], number of detected nucleotide alleles [N_alleles_], number of resulting amino acids [N_amino acid_], observed heterozygosity [H_obs_], expected heterozygosity [H_esp_] and haplotype diversity [h_div_].

Although fewer samples were available for the comparison, African leopards exhibited higher diversity (0.8617–0.9996) in the investigated TLR exons on the nucleotide level than cheetahs (0–0.9606). However, the diversity on the nucleotide level did not necessarily result in a higher amino acid diversity, e.g., the 23 nucleotide alleles for exon TLR4.2 (Table 1, Fig. 2) in the African leopards resulted in just four different amino acid sequences compared to the six nucleotide alleles in cheetahs that also resulted in four amino acid sequences.

The proportion of synonymous nucleotide alleles varied between TLR exons and species. The highest number of synonymous alleles were found in exon TLR4.2 for African leopards (Fig. 2) and exon TLR8 for cheetahs (Fig. 4). Furthermore, TLR8 was the least diverse exon in both species. Interestingly, TLR6 showed nucleotide alleles with a frameshift mutation in both leopards (two base pair deletion) and cheetahs (four base pair deletion) causing early stop codons (Fig. 3). We created three-dimensional models for the shortened polypeptides for both species using AlphaFold2^62^ to visualize the structural alteration. As per-residue confidence estimates, pLDDT (predicted Local Distance Difference Test) scores were extracted and included in the highest ranked structure (Supplementary Fig. 1). Homozygote individuals regarding the deletion in question occurred in both species and were likely unable to synthesize the full-length protein.

### Genetic diversity in TLR exons of historic cheetah samples

Historic cheetah samples showed additional nucleotide alleles (Figs. 1, 2, 3, 4) and historic haplotype diversity was always higher compared to modern Southern African cheetahs (Table 1). While no structure within the cheetah was apparent, unique alleles were found in all historic samples irrespective of their subspecies (Supplementary Table 2). In some cases, however, insufficient read coverage after mapping did not allow for accurate variant calling, and samples with less than 6 × coverage were removed from subsequent analyses. Especially, statements about historic diversity in TLR8 must be treated with caution, since less than half of all historic samples passed variant calling due to insufficient coverage.

### Phylogenetic structure of TLR2, TLR4, TLR6 and TLR8 in African leopards and cheetahs

Both species’ TLRs were phylogenetically separated at the nucleotide level and, to a lesser degree, at the amino acid level. In the case of TLR6, cheetah amino acid sequences were nested within sequences from African leopards (Fig. 3). While more basal nodes separating species were mostly well supported (bootstrap support > 80), internal nodes (within species) were predominantly uncertain, resulting in poorly resolved topologies (bootstrap support < 50).

### Episodic positive selection

In general, few sites of selection were detected (Supplementary Table 3). The codon-based analysis of selection signals in MEME only revealed four sites showing positive diversifying selection in African leopards and none in cheetahs. On the other hand, FEL indicated slightly more sites under positive diversifying selection in both species, notably in TLR6 (13 sites in leopards, 6 sites in cheetahs). Every TLR showed sites under purifying selection ranging from 5 sites in TLR4.2 to 22 sites in TLR6 in leopards, and from 1 site in TLR4.2 to 23 sites in TLR6 in cheetahs.

## Discussion

Resources for conservation are limited and studies on endangered species must meaningfully evaluate the standing genetic diversity. High levels of polymorphism in immune genes, especially in IIS-genes, directly affect a species’ ability to react to and counter infectious diseases. Consequently, a species’ resilience against diverse pathogens and its ability to resist future environmental changes are directly linked to genetic diversity. Leopards and cheetahs are threatened by human activity and several subspecies already face the consequences of genetic impoverishment and small population sizes^50,63^. Surviving in the Anthropocene means coping with strongly altered environments and living close to humans is inevitable, which increases the exposure risk to pathogens from life stock and domestic pets. To identify populations of concern, evaluating a species’ innate immunity is key to understanding its resilience to environmental change and maximizing conservation efforts. Within this study, we shed some light on the still poorly understood innate immunity of two large carnivores of the African Savana, African leopards and Southern African cheetahs, and provide a first assessment of genetic diversity in four selected TLR exons.

Leopards and cheetahs possess different levels of genetic diversity in the investigated TLR exons. Yet, they share some structural and haplotypic similarities. In direct comparison, modern African leopard’s TLR genes are much more diverse than those of modern Southern African cheetahs. This is especially true for TLR2 and TLR8; e. g., modern African leopards exhibit 32 different nucleotide alleles in TLR8, while modern Southern African cheetahs only exhibit one allele and historic cheetah only two (Table 1). Both species show comparatively higher variability in TLR2 and TLR6, which are known to form heterodimers essential for recognizing a large variety of bacterial PAMPs^7,64^. Leopards consistently showed higher variability than cheetahs. Interestingly, several TLR6 alleles in modern leopards and cheetahs showed reading frame shifts. Correspondingly, shortened polypeptides will be synthesized (Supplementary Fig. 1), similar to an already known reading frame shift in TLR2^65^, indicating a potential loss of function. In addition to TLR2-TLR6-heterodimers, TLR2 can also dimerize with TLR1, resulting in almost identical signal pathways, but also in slightly altered PAMP recognition^66^. Therefore, a potential loss of function in TLR6 might be compensated by increased diversity in TLR1 and should be considered in future studies. Both of our modern sample sets of Southern African cheetahs and African leopards included homozygote individuals that possessed those frame shift mutations in TLR6, which might indicate that deleterious alleles are already abundant in both species. Notably, similar deleterious alleles are absent in our historic cheetah sample set, which could indicate a recent accumulation of harmful mutations in TLR6 in modern Southern African cheetahs. Unfortunately, we lack historic data for comparison in African leopards. In addition, TLR6 was the only TLR that showed some sites under positive diversifying selection in both species and a more extensive historic sample set might help to understand its evolutionary trend in the future.

In general, our historic cheetah samples are more heterozygous compared to modern Southern African cheetahs. This holds true even excluding other subspecies, the three historic Southern African cheetahs were more heterozygous (TLR2, TLR6) and exhibited more unique haplotypes (e.g., 3 vs. 2 in TLR2; 6 vs. 3 in TLR6) than our 49 contemporary samples (Table 1). We are aware that Illumina short-read data is not directly comparable to PacBio long-read data and variant calling from short reads is more difficult due to the additional phasing. Therefore, we discarded samples with less than 6× coverage and manually verified each allele. Still, we have identified new unique loci present in different subspecies of historic cheetahs that are absent in modern South African individuals.

In comparison, TLR4.2 and TLR8 seem to be less diverse than the other exons of TLR2 and TLR6 in historic and modern cheetahs. This supports previous studies that identified TLR4 and TLR8 as more conserved among non-closely related species^67,68^. However, as both TLR exons were more difficult to amplify in modern samples and also less covered in the historic samples, we would need to investigate more individuals to understand the functional evolution of TLR4 and TLR8 in the species.

Historic cheetah samples exhibited consistently lower haplotype diversity in the investigated TLR genes compared to modern African leopards, but higher than modern Southern African cheetah. Therefore, loss in genetic diversity and low heterozygosity in contemporary Southern African cheetahs could be more recent and the result of a population decline within the last 150 years. Even if only this cheetah subspecies is considered, the historic genetic diversity is visible higher. It is known that increasing human activity in Africa in the previous century reduced the numbers of large carnivores drastically^69,70^, and both leopards and cheetahs became extinct in large parts of their former ranges^44,48^. However, the African leopard appeared to be highly diverse regarding the investigated TLRs (this study) and on the genomic level^53^ and was seemingly far less affected by human activity. While we chose both species due to their similar lifestyle and sympatric distribution in Savanna habitats, there are still noticeable differences that might explain a deviating innate immune response gene diversity. Cheetahs are highly specialized carnivores that only occur in open habitats, African leopards, on the other hand, are less tied to a specific biome. Except for desserts, they occur in mountain ridges and open grasslands as well as in tropical forests and marshlands^71^. Therefore, current leopard populations are larger and less fragmented than modern cheetah populations^44,72^. Habitat diversity and population size might explain the higher genetic diversity in the leopards’ TLR genes because they are likely more exposed to different and more diverse pathogens. Additionally, persecution might threaten cheetahs more because of their diurnal lifestyle and the recently increasing illegal pet trade^73^.

Due to easy accessibility, most immunological studies on cheetahs and African leopards only included their Southern and Southeastern African distribution (this study included). Hence, only two classical cheetah subspecies (*A. j. jubatus* and *A. j. raineyi*) and less than half of the African leopard’s current distribution are covered. Yet, conclusions about the immune response in both species should consider subspecies and local populations because the imposed environmental pressure might differ significantly; a problem only to be solved by extended modern and historic sample sets throughout both species’ distribution. By extending our sampling to the other cheetah subspecies and also including historic material, we aimed to account for the exposure to different environmental pressures. Although, modern Southern African cheetahs exhibit lower heterozygosity and lower diversity in TLR exons than African leopards, this might not be representative of TLR genes as a whole in the species. We mostly investigated anti-bacterial TLRs that are known to differ, e.g., in selection patterns, from virus detecting TLRs^74,75^. Furthermore, anti-viral TLRs are assumed to be under stronger evolutionary constrain than other TLR-families^76,77^. Nevertheless, it was shown that the constitutive innate immune response in Southern African cheetahs is stronger than in sympatric leopards^78^. Thus, relevant levels of genetic diversity might occur in other IIS-genes. However, genetic deprivation might already similarly affect African leopards despite apparently high genetic diversity. Moreover, MHC diversity in cheetahs is not as low as previously expected^58,59,61^ and the species’ health seems not to be affected at all in the wild^78,79^. This hints at a sufficiently high adaptive AIS activity in cheetahs, which might enable the species to cope with its low diversity in specific IIS genes^80^. Further studies should investigate co-evolution between more IIS and AIS genes in cheetahs and leopards and include additional historic data.

In conclusion, like all large carnivores, leopards and cheetahs in Africa are threatened less by their genetic heritage but more by recent habitat loss and persecution. So far, the genetic diversity of African leopards does not appear to have been greatly affected by human activity, while the genetic impoverishment of cheetahs is the direct result of the recent population decline in this highly specialized species. Our study observed a drastic reduction in genetic diversity and heterozygosity in four TLR exons of modern Southern African cheetahs, which may worsen this subspecies’ resilience to future infectious diseases. However, the health of the subspecies does not yet appear to be affected by its reduced genetic diversity. Still, the growing human-livestock-wildlife interface will further increase the risk of infectious diseases for wild populations.

## Material and methods

### Sample collection

To study the diversity in TLR genes in big cats, we used 61 modern Southern African cheetah samples (*Acinonyx jubatus jubatus*) from the South African National Biodiversity Institute (SANBI) Biobanks (Pretoria, South Africa; approved project number P2021/12) that had been collected from wild populations between 1998 and 2014 during different translocation and monitoring projects, including 14 individuals from Botswana, 12 from Namibia and 35 from South Africa. To retrieve immune gene diversity information from all cheetah subspecies we added 15 samples from museums’ collections, including five Asiatic cheetahs (*A. j. venaticus*), one East African cheetah (*A. j. raineyi*), four Northeast African cheetahs (*A. j. soemmeringii*), three Southern African cheetahs and three West African cheetahs (*A. j hecki*). In addition, we included short-read data of 41 previously sequenced African leopards (*Panthera pardus pardus*)^49^, compromising four individuals from Ghana, eight from Namibia, 14 from Tanzania, and 15 from Zambia. For detailed sample information see Supplementary Table 1. Samples collected after 1975 were imported under the following CITES permits: AT 16-E-0753, 16SG006329CR, 15JP001990/TE, 11US761881/9, AT 15-E-1769, D79/DFF or transferred between CITES-registered institutions (Supplementary Table 4).

Genomic DNA was extracted from modern samples using the *Quick*-DNA Miniprep Plus Kit (Zymo Research, Irvine, California, USA) and a DNA salting out method^81^ for historic samples. Historic samples were rehydrated in nuclease-free water for 24 h in an attempt to remove potential secondary preservatives before DNA extraction.

### Library preparation and next-generation sequencing

In preparation for long-read amplicon sequencing (LRS) we created primer sets to investigate the five exons of the selected toll-like receptor genes TLR2, TLR4, TLR6 and TLR8 (Supplementary Table 5) using the available cheetah genome from NCBI (GCF_003709585.1)^82^ with primer3^83^. We amplified our modern *A. j. jubatus* samples using long-range Polymerase Chain Reaction and targeted specific primers tailed with M13 adapter sequences. Per individual sample and exon, we used a total reaction volume of 10 µl consisting of 5 µl GoTaq® Long PCR Master Mix (Promega, Madison, Wisconsin, USA), 2.5 µl ddH_2_O, 0.5 µl of both forward and reverse primers (10 µM) and 1.5 µl of template DNA (50–250 ng). The following PCR protocol was used: Initial denaturation at 95 °C for 2 min following 35 cycles (denaturation at 95 °C for 30 s, annealing at 57 °C for 30 s and elongation at 72 °C for 5 min), and a final elongation at 72 °C for 10 min. Amplicons containing PacBio M13 adaptor sequences (Pacific Biosciences of California, Menlo Park, California, USA) were sent to Inqaba Biotechnical Industries (Pretoria, South Africa) for indexing. A PacBio circular consensus sequencing (CCS) library was prepared using the SMRTbell® express template prep kit 2.0 (PacBio), indexed and the resulting amplicons were sequenced on the PacBio Sequel IIe platform (PacBio).

Whole genomes of historic cheetahs (*A. jubatus ssp.*) were sequenced as part of a larger project, and in-depth analyses are still ongoing due to necessary re-sequencing of some particularly difficult samples. In brief, we prepared 150 bp paired-end libraries for short-read sequencing using the NEBNext® Ultra™ II DNA Library Prep Kit for Illumina® (New England Biolabs, Ipswich, Massachusetts, USA) with varying insert sizes depending on DNA quality. All libraries were sequenced on the NovaSeq 6000 platform (Illumina, San Diego, California, USA) at Novogene (Cambridge, England, UK).

### Data processing

Long-read sequences were received as high fidelity (HIFI) consensus reads and no additional trimming/filtering was necessary. Short reads were trimmed with fastp version 0.20.1^84^ and filtered with base correction enabled and a low complexity filter. All sequencing adapters and polyG tails at the end of reads were removed and a sliding window of 4 bp was applied to detect poor-quality regions (Phred score < 15). Short reads were discarded if they were shorter than 36 bp, had > 40% low-quality bases, or had more than five undetermined bases. We mapped our long reads to the TLR reference sequences derived from the cheetah assembly (GCF_003709585.1) using bwa-mem^85^ and removed duplicates with Picard version 2.22.3^86^. The variant calling of both alleles was performed with BCFtools mpileup version 1.9^87^ (flags: -s LowQual -e '%QUAL < 20 || DP > 10') in two consecutive steps using the first called consensus as reference for the second variant. The short reads were first mapped to the TLR consensus sequences using bwa-mem and sorted by name using SAMtools sort^88^. Unmapped reads were removed, and the reads in the resulting mapping file were converted to a new fastq file using SAMtools view. The reduced reads were mapped a second time using bowtie2 version 2.4.5^89^ without any clipping (flags: -end-to-end -x -S) to avoid miss called Single Nuclear Polymorphisms (SNPs) due to over-representation caused by falsely clipped reads. Variant calling followed the same steps described for the long reads above. The script used for mapping and variant calling is provided in the Supplementary material. Insertions and deletions (indels) were curated manually, and each called SNP was re-checked for validity using Tablet version 1.21.02.08^90^.

We used the PHASE function implemented in DnaSP v.6.12.03^91^ to derive the alleles of the short-read consensus sequences with a threshold of 0.6, allowing for recombination. No additional phasing for long-read sequences was needed. All further analyses were performed using phased allele sequences.

### Comparative analysis

DNA sequences were aligned using the aligner integrated in AliView^92^. Identical sequences were removed keeping only a single sequence per allele and translated into amino acid sequences. Amino acid and underlying nucleotide alleles were named according to the naming scheme described in Supplementary Fig. 2. Maximum likelihood phylogenies were generated from aligned DNA sequences and the resulting amino acid sequences of each TLR exon using IQ-TREE version 1.6.12^93^. TLR sequences of the spotted hyena (*Crocuta crocuta*) (GCA_008692635.1 BGI_CrCroc_1.0) and striped hyena (*Hyena hyena*) (GCA_003009895.1 ASM300989v1) were used as an outgroup. We used the term “allele” to refer to full-length allele sequences defined by LRS and not to individual SNPs within the exon sequence.

SNP-sites were investigated for recombination with the Genetic Algorithm for Recombination Detection tool (GARD)^94^. We used the resulting NEXUS files to detect episodic positive selection based on the Mixed Effects Model of Evolution (MEME)^95^, both implemented in the online tool datamonkey^96^.

### Ethics approval and consent to participate

The study was approved by the institutional review board of the South African National Biodiversity Institute (SANBI) with the project number P2021/12. No animal experiments were conducted in this study. No human participants, tissue, or data were used in this study.

### Supplementary Information

Supplementary Information.

## Data Availability

All data generated or analyzed during this study are included within this article’s Supplementary material. Additionally, the newly generated TLR sequencing reads are uploaded as FASTQ files to NCBI (PRJNA1005947) and the corresponding TLR 2, 4, 6, and 8 alignments for both species are available on the Phaidra public repository: https://doi.org/10.34876/9KFD-2A38.
