# Supplementary material for: Diversity of selected toll-like receptor genes in cheetahs (Acinonyx jubatus) and African leopards (Panthera pardus pardus)
Source: Sci Rep. 2024 Feb 14;14:3756. doi: 10.1038/s41598-024-54076-y (PMC10866938; doi:10.1038/s41598-024-54076-y)
Supplement: Supplementary file 1 — Supplementary Information. [file 41598_2024_54076_MOESM1_ESM.pdf]

Supplementary material

Supplementary Table 1: Complete list of all individual leopard and cheetah samples used within this study with detailed information (including species, assigned subspecies, geographical origin, collection date and institution of origin), IDs starting with AJ are from museum collections. Amplified TLRs indicated by X.

ID	Species	Subspecies	Country	Collection date	Institution	TLR 2	TLR 4.2	TLR 6	TLR 8
506	Cheetah	*A. j. jubatus*	South Africa	13.07.1999	SANBI*[1]	X	X	X	X
507	Cheetah	*A. j. jubatus*	South Africa	14.07.1999	SANBI	X	X	X	X
523	Cheetah	*A. j. jubatus*	South Africa	31.08.1999	SANBI	X	X	X	X
527	Cheetah	*A. j. jubatus*	South Africa	15.09.1999	SANBI	X	X	X	X
589	Cheetah	*A. j. jubatus*	South Africa	01.04.2000	SANBI	X	X	X	X
655	Cheetah	*A. j. jubatus*	South Africa	29.05.2000	SANBI	X	X	X	X
678	Cheetah	*A. j. jubatus*	South Africa	08.06.2000	SANBI	X	X	X	X
680	Cheetah	*A. j. jubatus*	South Africa	09.06.2000	SANBI	X	X	X	X
1139	Cheetah	*A. j. jubatus*	South Africa	08.08.2001	SANBI	X	X	X	X
1234	Cheetah	*A. j. jubatus*	South Africa	26.01.1998	SANBI	X	X	X	X
1403	Cheetah	*A. j. jubatus*	South Africa	02.08.2002	SANBI	X	X	X	X
1448	Cheetah	*A. j. jubatus*	South Africa	13.09.2002	SANBI	X	X	X	
1903	Cheetah	*A. j. jubatus*	South Africa	14.11.2002	SANBI	X	X	X	X
1914	Cheetah	*A. j. jubatus*	South Africa	27.11.2002	SANBI	X	X	X	X
1949	Cheetah	*A. j. jubatus*	South Africa	03.01.2003	SANBI	X	X	X	X
1968	Cheetah	*A. j. jubatus*	South Africa	07.02.2003	SANBI	X	X	X	X
3004	Cheetah	*A. j. jubatus*	South Africa	19.03.2003	SANBI	X	X	X	X
3006	Cheetah	*A. j. jubatus*	South Africa	27.03.2003	SANBI	X	X	X	X
3024	Cheetah	*A. j. jubatus*	South Africa	24.04.2003	SANBI	X	X	X	X
3835	Cheetah	*A. j. jubatus*	South Africa	27.02.2004	SANBI	X	X	X	X
4683	Cheetah	*A. j. jubatus*	South Africa	02.02.2006	SANBI		X		X
5666	Cheetah	*A. j. jubatus*	South Africa	14.09.2005	SANBI	X	X	X	X
10579	Cheetah	*A. j. jubatus*	South Africa	19.11.2007	SANBI	X	X		
10878	Cheetah	*A. j. jubatus*	South Africa	29.01.2008	SANBI	X	X	X	X
11775	Cheetah	*A. j. jubatus*	South Africa	22.07.2008	SANBI	X	X	X	X
14170	Cheetah	*A. j. jubatus*	South Africa	31.08.2009	SANBI	X	X	X	X
15039	Cheetah	*A. j. jubatus*	South Africa	before 2015	SANBI	X	X	X	X
25182	Cheetah	*A. j. jubatus*	South Africa	23.01.2013	SANBI	X	X	X	X
25344	Cheetah	*A. j. jubatus*	Namibia	08.08.2011	SANBI	X	X	X	X
25345	Cheetah	*A. j. jubatus*	Namibia	08.08.2011	SANBI	X		X	X
25346	Cheetah	*A. j. jubatus*	Namibia	2011	SANBI	X	X	X	X
29341	Cheetah	*A. j. jubatus*	Namibia	09.08.2011	SANBI	X	X	X	X
29366	Cheetah	*A. j. jubatus*	Namibia	12.06.2012	SANBI	X	X	X	X

29367	Cheetah	*A. j. jubatus*	Namibia	12.06.2012	SANBI	X	X	X	X
29376	Cheetah	*A. j. jubatus*	Namibia	11.06.2012	SANBI	X	X	X	X
32604	Cheetah	*A. j. jubatus*	South Africa	11.04.2014	SANBI	X		X	
32607	Cheetah	*A. j. jubatus*	South Africa	29.04.2014	SANBI	X	X	X	
33821	Cheetah	*A. j. jubatus*	South Africa	08.05.2014	SANBI	X		X	
34309	Cheetah	*A. j. jubatus*	Namibia	08.07.2014	SANBI	X	X	X	
34311	Cheetah	*A. j. jubatus*	Namibia	08.07.2014	SANBI	X		X	X
34314	Cheetah	*A. j. jubatus*	Namibia	08.07.2014	SANBI				
34327	Cheetah	*A. j. jubatus*	Namibia	03.07.2014	SANBI				
34335	Cheetah	*A. j. jubatus*	Namibia	16.06.2014	SANBI	X	X		X
35865	Cheetah	*A. j. jubatus*	South Africa	02.07.2014	SANBI				
35895	Cheetah	*A. j. jubatus*	South Africa	07.07.2014	SANBI			X	
35988	Cheetah	*A. j. jubatus*	South Africa	06.08.2014	SANBI	X		X	
35989	Cheetah	*A. j. jubatus*	South Africa	23.07.2014	SANBI	X	X	X	X
53855	Cheetah	*A. j. jubatus*	Botswana	2005	SANBI	X		X	
53856	Cheetah	*A. j. jubatus*	Botswana	2005	SANBI	X	X	X	
53857	Cheetah	*A. j. jubatus*	Botswana	2005	SANBI	X	X	X	
53858	Cheetah	*A. j. jubatus*	Botswana	2005	SANBI	X	X	X	X
53859	Cheetah	*A. j. jubatus*	Botswana	2005	SANBI	X	X	X	X
53860	Cheetah	*A. j. jubatus*	Botswana	2005	SANBI			X	X
53861	Cheetah	*A. j. jubatus*	Botswana	2005	SANBI				
53862	Cheetah	*A. j. jubatus*	Botswana	2005	SANBI	X			
53863	Cheetah	*A. j. jubatus*	Botswana	2005	SANBI				
53864	Cheetah	*A. j. jubatus*	Botswana	2005	SANBI			X	
53865	Cheetah	*A. j. jubatus*	Botswana	2005	SANBI			X	
53866	Cheetah	*A. j. jubatus*	Botswana	2005	SANBI			X	
53867	Cheetah	*A. j. jubatus*	Botswana	2005	SANBI				
53868	Cheetah	*A. j. jubatus*	Botswana	2005	SANBI				
AJ_G2	Cheetah	*A. j. venaticus*	Iran	before 1970	MI*[2]	X	X	X	X
AJ013	Cheetah	*A. j. jubatus*	Angola	1936	PCM*[3]	X	X	X	X
AJ064	Cheetah	*A. j. venaticus*	Iran	before 2000	CE3c*[4]	X	X	X	X
AJ078	Cheetah	*A. j. hecki*	Algeria	1943	CSIC*[5]	X		X	
AJ079	Cheetah	*A. j. venaticus*	Afghanistan	1949	ZMUC*[6]	X	X	X	X
AJ085	Cheetah	*A. j. jubatus*	Namibia	1924	AMKWT*[7]	X	X	X	
AJ095	Cheetah	*A. j. jubatus*	Zambia	1877	NHM*[8]	X	X	X	
AJ119	Cheetah	*A. j. raineyi*	Tanzania	1909	NMB*[9]	X	X	X	
AJ139	Cheetah	*A. j. venaticus*	Turkmenistan	1933	ZIRAS*[10]	X		X	
AJ178	Cheetah	*A. j. venaticus*	Iraq	1912	TAU*[11]	X			
AJ271	Cheetah	*A. j. hecki*	Nigeria	1970	YGRM*[12]	X	X	X	
AJ305	Cheetah	*A. j. soemmeringii*	Somalia	2007	CE3c	X	X	X	X
AJ306	Cheetah	*A. j. soemmeringii*	Somalia	2007	CE3c	X	X	X	X
AJ354	Cheetah	*A. j. hecki*	Libya	before 1970	NMW*[13]	X	X	X	X
AJ399	Cheetah	*A. j. soemmeringii*	Ethiopia	1930	DECAN*[14]	X	X	X	
2469	Leopard	*P. p. pardus*	Zambia	1995	PP*[15]	X	X	X	X

3241	Leopard	*P. p. pardus*	Zambia	1995	PP	X	X	X	X
3243	Leopard	*P. p. pardus*	Tanzania	1995	PP	X	X	X	X
3244	Leopard	*P. p. pardus*	Tanzania	1995	PP	X	X	X	
4343	Leopard	*P. p. pardus*	Tanzania	1993	PP	X	X	X	X
4346	Leopard	*P. p. pardus*	Tanzania	1993	PP	X	X	X	
4352	Leopard	*P. p. pardus*	Tanzania	1994	PP	X	X	X	
4354	Leopard	*P. p. pardus*	Tanzania	1994	PP	X	X	X	X
4443	Leopard	*P. p. pardus*	Tanzania	1995	PP	X	X		
5180	Leopard	*P. p. pardus*	Tanzania	1996	PP	X		X	
5181	Leopard	*P. p. pardus*	Tanzania	1996	PP		X	X	
5519	Leopard	*P. p. pardus*	Tanzania	1997	PP	X	X	X	
5520	Leopard	*P. p. pardus*	Tanzania	1997	PP	X	X		
5521	Leopard	*P. p. pardus*	Tanzania	1996	PP	X	X	X	
5522	Leopard	*P. p. pardus*	Tanzania	1997	PP	X	X		
5525	Leopard	*P. p. pardus*	Tanzania	1997	PP	X	X	X	
6342	Leopard	*P. p. pardus*	Zambia	NA	PP	X	X	X	X
6344	Leopard	*P. p. pardus*	Zambia	NA	PP	X	X	X	X
6346	Leopard	*P. p. pardus*	Zambia	NA	PP	X	X	X	X
6348	Leopard	*P. p. pardus*	Zambia	NA	PP	X	X	X	
6349	Leopard	*P. p. pardus*	Zambia	NA	PP	X	X	X	X
6351	Leopard	*P. p. pardus*	Zambia	NA	PP	X	X	X	X
6353	Leopard	*P. p. pardus*	Zambia	NA	PP	X	X	X	X
6354	Leopard	*P. p. pardus*	Zambia	NA	PP	X	X	X	X
6355	Leopard	*P. p. pardus*	Zambia	NA	PP	X	X	X	
6356	Leopard	*P. p. pardus*	Zambia	NA	PP	X	X	X	
6357	Leopard	*P. p. pardus*	Zambia	NA	PP	X	X	X	X
6358	Leopard	*P. p. pardus*	Zambia	NA	PP	X	X	X	X
6359	Leopard	*P. p. pardus*	Zambia	NA	PP	X	X	X	X
7246	Leopard	*P. p. pardus*	Ghana	1998	PP	X	X	X	X
7547	Leopard	*P. p. pardus*	Ghana	1998	PP	X	X	X	X
7548	Leopard	*P. p. pardus*	Ghana	1998	PP		X	X	X
7549	Leopard	*P. p. pardus*	Ghana	1998	PP			X	X
7934	Leopard	*P. p. pardus*	Namibia	1998	PP	X	X	X	X
7935	Leopard	*P. p. pardus*	Namibia	1998	PP	X	X	X	
7936	Leopard	*P. p. pardus*	Namibia	1998	PP	X	X	X	X
7937	Leopard	*P. p. pardus*	Namibia	1998	PP	X		X	
7938	Leopard	*P. p. pardus*	Namibia	1997	PP	X	X	X	
7939	Leopard	*P. p. pardus*	Namibia	1997	PP	X	X	X	
7940	Leopard	*P. p. pardus*	Namibia	1997	PP	X	X	X	X
7941	Leopard	*P. p. pardus*	Namibia	1998	PP	X	X	X	X
7942	Leopard	*P. p. pardus*	Namibia	1997	PP	X	X	X	X

*[1] South African National Biodiversity Institute (SANBI) Biobankes, *[2] Museum of Iran, Iran, *[3] Powell-Cotton Museum Quex Park, UK, *[4] CE3c; Breeding Centre for Endangered Arabian Wildlife, UAE, *[5] CSIC Donana – Seville HQ, Spain, *[6] Zoological Museum Univ.Copenhagen, Denmark, *[7] Amathole Museum King Williams Town, South Africa, *[8] Natural History Museum London, UK, *[9] Museum für Naturkunde Berlin, Germany, *[10] Zool. Institute Russian Acad. Sciences St. Petersburg, Russia, *[11] Tel Aviv University Zoological Museum, Israel, *[12] Yankari Game Reserve Museum, Nigeria, *[13] Naturhistorisches Museum Wien, Austria, *[14] DECAN refuge, Djibouti, *[15] Patrícia Pečnerová et al. 2021 https://doi.org/10.1016/j.cub.2021.01.064

Supplementary Table 2: TLR diversity of historic cheetah samples; including sample ID [ID], assigned cheetah subspecies [SSP], country of the sample's origin [origin], collection date and specified TLR exon. For each sample both nucleotide alleles or homozygosity are shown, and differences in resulting amino acids are indicated by color differences. Blancs indicate missing data.

ID	SSP	origin	collection date	TLR2	TLR4-2	TLR6	TLR8
AJ078	*A. j. hecki*	Algeria	1943	aaAcJu-TLR2*01_01		aaAcJu-TLR6*08_01	
				aaAcJu-TLR2*03_01		aaAcJu-TLR6*08_02	
AJ271	*A. j. hecki*	Nigeria	1970	aaAcJu-TLR2*01_01	aaAcJu-TLR4.2*01_02	aaAcJu-TLR6*13_01	
				aaAcJu-TLR2*01_02	aaAcJu-TLR4.2*03_01	aaAcJu-TLR6*14_01	
AJ354	*A. j. hecki*	Libya	before 1940	aaAcJu-TLR2*01_01	aaAcJu-TLR4.2*03_01	aaAcJu-TLR6*01_02	aaAcJu_TLR8*01_01
				aaAcJu-TLR2*01_02	>>>homozygot<<<	aaAcJu-TLR6*05_01	>>>homozygot<<<
AJ013	*A. j. jubatus*	Angola	1936	aaAcJu-TLR2*01_01	aaAcJu-TLR4.2*01_01	aaAcJu-TLR6*04_01	aaAcJu_TLR8*01_01
				aaAcJu-TLR2*03_01	>>>homozygot<<<	aaAcJu-TLR6*05_01	aaAcJu_TLR8*01_02
AJ085	*A. j. jubatus*	Namibia	1924	aaAcJu-TLR2*01_01	aaAcJu-TLR4.2*01_01	aaAcJu-TLR6*11_01	
				aaAcJu-TLR2*01_02	aaAcJu-TLR4.2*04_01	aaAcJu-TLR6*11_02	
AJ095	*A. j. jubatus*	South Africa	1882	aaAcJu-TLR2*01_03	aaAcJu-TLR4.2*01_01	aaAcJu-TLR6*11_03	
				aaAcJu-TLR2*05_01	>>>homozygot<<<	aaAcJu-TLR6*12_01	
AJ119	*A. j. raineyi*	Tanzania	1909	aaAcJu-TLR2*01_01	aaAcJu-TLR4.2*01_01	aaAcJu-TLR6*05_01	
				>>>homozygot<<<	aaAcJu-TLR4.2*03_02	>>>homozygot<<<	
AJ305	*A. j. soemmeringii*	Somalia	2001	aaAcJu-TLR2*01_01	aaAcJu-TLR4.2*03_01	aaAcJu-TLR6*15_01	aaAcJu_TLR8*01_01
				aaAcJu-TLR2*01_02	>>>homozygot<<<	aaAcJu-TLR6*05_01	>>>homozygot<<<
AJ306	*A. j. soemmeringii*	Somalia	2001	aaAcJu-TLR2*01_01	aaAcJu-TLR4.2*01_01	aaAcJu-TLR6*15_01	aaAcJu_TLR8*01_01
				aaAcJu-TLR2*01_04	aaAcJu-TLR4.2*03_01	aaAcJu-TLR6*05_02	>>>homozygot<<<
AJ399	*A. j. soemmeringii*	Ethiopia	1930	aaAcJu-TLR2*01_01	aaAcJu-TLR4.2*01_01	aaAcJu-TLR6*01_03	
				aaAcJu-TLR2*08_01	aaAcJu-TLR4.2*03_01	aaAcJu-TLR6*05_03	
AJ064	*A. j. venaticus*	Iran	before 2000	aaAcJu-TLR2*04_01	aaAcJu-TLR4.2*01_01	aaAcJu-TLR6*06_01	aaAcJu_TLR8*01_02
				aaAcJu-TLR2*04_02	>>>homozygot<<<	aaAcJu-TLR6*07_01	>>>homozygot<<<
AJ079	*A. j. venaticus*	Afghanistan	1949	aaAcJu-TLR2*01_01	aaAcJu-TLR4.2*01_01	aaAcJu-TLR6*09_01	aaAcJu_TLR8*01_02
				>>>homozygot<<<	>>>homozygot<<<	aaAcJu-TLR6*10_01	>>>homozygot<<<
AJ139	*A. j. venaticus*	Turkmenistan	1934	aaAcJu-TLR2*01_01		aaAcJu-TLR6*08_03	
				aaAcJu-TLR2*06_01		aaAcJu-TLR6*08_04	
AJ178	*A. j. venaticus*	Iraq	1912	aaAcJu-TLR2*07_01			
				>>>homozygot<<<			
AJ_G2	*A. j. venaticus*	Iran	before 2000	aaAcJu-TLR2*09_01	aaAcJu-TLR4.2*01_01	aaAcJu-TLR6*04_02	aaAcJu_TLR8*01_01
				aaAcJu-TLR2*09_02	>>>homozygot<<<	aaAcJu-TLR6*05_01	>>>homozygot<<<

Supplementary Table 3: Sites under selection in cheetahs and leopards. Information includes TLR exon [TLR], site position [Codon], type of selection detected [Selection type] and used method [Method].

TLR	Species	Codon	Selection type	Method
TLR2	Cheetah	531	purifying selection	FEL
TLR2	Cheetah	745	purifying selection	FEL
TLR2	Leopard	63	deversifying positive	FEL
TLR2	Leopard	542	deversifying positive	FEL
TLR2	Leopard	677	deversifying positive	FEL/MEME
TLR2	Leopard	733	deversifying positive	FEL
TLR2	Leopard	33	purifying selection	FEL
TLR2	Leopard	42	purifying selection	FEL
TLR2	Leopard	317	purifying selection	FEL
TLR2	Leopard	545	purifying selection	FEL
TLR2	Leopard	569	purifying selection	FEL
TLR2	Leopard	642	purifying selection	FEL
TLR2	Leopard	693	purifying selection	FEL
TLR2	Leopard	708	purifying selection	FEL
TLR2	Leopard	721	purifying selection	FEL
TLR4.2	Cheetah	678	purifying selection	FEL
TLR4.2	Leopard	214	purifying selection	FEL
TLR4.2	Leopard	345	purifying selection	FEL
TLR4.2	Leopard	467	purifying selection	FEL
TLR4.2	Leopard	563	purifying selection	FEL

TLR4.2	Leopard	622	purifying selection	FEL
TLR6	Cheetah	563	deversifying positive	FEL
TLR6	Cheetah	658	deversifying positive	FEL
TLR6	Cheetah	661	deversifying positive	FEL
TLR6	Cheetah	663	deversifying positive	FEL
TLR6	Cheetah	728	deversifying positive	FEL
TLR6	Cheetah	729	deversifying positive	FEL
TLR6	Cheetah	463	purifying selection	FEL
TLR6	Cheetah	494	purifying selection	FEL
TLR6	Cheetah	568	purifying selection	FEL
TLR6	Cheetah	626	purifying selection	FEL
TLR6	Cheetah	643	purifying selection	FEL
TLR6	Cheetah	648	purifying selection	FEL
TLR6	Cheetah	651	purifying selection	FEL
TLR6	Cheetah	660	purifying selection	FEL
TLR6	Cheetah	662	purifying selection	FEL
TLR6	Cheetah	695	purifying selection	FEL
TLR6	Cheetah	699	purifying selection	FEL
TLR6	Cheetah	707	purifying selection	FEL
TLR6	Cheetah	710	purifying selection	FEL
TLR6	Cheetah	712	purifying selection	FEL
TLR6	Cheetah	719	purifying selection	FEL
TLR6	Cheetah	720	purifying selection	FEL
TLR6	Cheetah	723	purifying selection	FEL
TLR6	Cheetah	730	purifying selection	FEL

TLR6	Cheetah	732	purifying selection	FEL
TLR6	Cheetah	733	purifying selection	FEL
TLR6	Cheetah	737	purifying selection	FEL
TLR6	Cheetah	738	purifying selection	FEL
TLR6	Cheetah	761	purifying selection	FEL
TLR6	Leopard	458	purifying selection	FEL
TLR6	Leopard	463	purifying selection	FEL
TLR6	Leopard	522	purifying selection	FEL
TLR6	Leopard	536	purifying selection	FEL
TLR6	Leopard	566	purifying selection	FEL
TLR6	Leopard	568	purifying selection	FEL
TLR6	Leopard	596	purifying selection	FEL
TLR6	Leopard	632	purifying selection	FEL
TLR6	Leopard	648	purifying selection	FEL
TLR6	Leopard	650	purifying selection	FEL
TLR6	Leopard	651	purifying selection	FEL
TLR6	Leopard	660	purifying selection	FEL
TLR6	Leopard	662	purifying selection	FEL
TLR6	Leopard	673	purifying selection	FEL
TLR6	Leopard	699	purifying selection	FEL
TLR6	Leopard	719	purifying selection	FEL
TLR6	Leopard	720	purifying selection	FEL
TLR6	Leopard	723	purifying selection	FEL
TLR6	Leopard	730	purifying selection	FEL
TLR6	Leopard	732	purifying selection	FEL

TLR6	Leopard	435	deversifying positive	FEL/MEME
TLR6	Leopard	452	deversifying positive	FEL/MEME
TLR6	Leopard	459	deversifying positive	FEL
TLR6	Leopard	461	deversifying positive	FEL
TLR6	Leopard	468	deversifying positive	FEL
TLR6	Leopard	543	deversifying positive	FEL
TLR6	Leopard	563	deversifying positive	FEL
TLR6	Leopard	576	deversifying positive	FEL
TLR6	Leopard	584	deversifying positive	FEL
TLR6	Leopard	595	deversifying positive	FEL
TLR6	Leopard	658	deversifying positive	FEL
TLR6	Leopard	661	deversifying positive	FEL
TLR6	Leopard	663	deversifying positive	FEL
TLR8	Leopard	142	purifying selection	FEL
TLR8	Leopard	184	purifying selection	FEL
TLR8	Leopard	370	purifying selection	FEL
TLR8	Leopard	432	purifying selection	FEL
TLR8	Leopard	435	purifying selection	FEL
TLR8	Leopard	563	purifying selection	FEL
TLR8	Leopard	581	purifying selection	FEL
TLR8	Leopard	673	purifying selection	FEL
TLR8	Leopard	927	purifying selection	FEL
TLR8	Leopard	1031	purifying selection	FEL
TLR8	Leopard	658	deversifying positive	FEL/MEME

Supplementary Table 4: CITES-registered institutions within this study and their registration numbers.

Institution	**CITES Number**
Amathole Museum, King William's Town, South Africa	**ZA020**
Botswana National Museum, Gaborone, Botswana	**BW004**
cE3c – Centre for Ecology, Evolution and Environmental Changes, Faculdade de Ciências da Universidade de Lisboa, Portugal	**PT004**
Field Museum of Natural History, Chicago, USA	**US012**
Forschungsinstitut für Wildtierkunde und Ökologie, Vetmeduni Vienna, Österreich	**AT031**
Harvard University Museum of Comparative Zoology,Cambridge, 02138	**US051**
Koret School Veterinary Medicine, Hebrew University of Jerusalem, Israel	**IL002**
Kwa-Zulu Natal Museum, Pietermaritzburg, South Africa	**ZA025**
Leiden University, Institute of Biology Dept Integrative Zoology, Leiden, Netherlands	**NL001**
Museo Civico di Storia Naturale Giacomo Doria, Genova, Italy	**IT019**
Museum für Naturkunde Berlin, Berlin, Germany	**DE203-06**
Museum National d'Histoire Naturelle, Paris, France	**FR75A**
Museum of Evolution, Uppsala, Sweden	**SE010**
National Zoological Gardens of South Africa, Pretoria, South Africa	**ZA034**
Natural History Museum "La Specola", Florence, Italy	**IT008**
Natural History Museum Denmark, Copenhagen, Denmark	**DK003**
Natural History Museum of Geneva, Switzerland	**CH004**
Natural History Museum, London, England	**GB001**
Naturhistorisches Museum Basel, Switzerland	**CH002**
Naturmuseum Sankt Gallen, Switzerland	**CH033**
Real jardín botánico Consejo superior de investigaciones cientificas, Madrid, Spain	**ES001**
Tel Aviv University, Israel	**IL001**
Zoologisches Museum der Universität Zürich, Switzerland	**CH005**

Supplementary Table 5: Primers used in this study including target information, orientation, sequence, amplicon length, annealing temperature and GC-content.

Target	Orientation	Sequence	Lenght [bp]	Annealing temperatur [C°]	GC-content [%]
TLR2 (exon)	forward	GCCGCTCTGTTATTTCCAGG	3713	58.98	55
TLR2 (exon)	reverse	AAAGACCACCACCAGACCAA		59.08	50
TLR4 (exon 2)	forward	AAGTGCTCTCATGGAAGCCT	4791	59.01	50
TLR4 (exon 2)	reverse	TGATTTCACTAGGGCCCAGG		59.08	55
TLR6 (exon)	forward	AAGGTCTGTATTGCCTCCCC	2979	59.08	55
TLR6 (exon)	reverse	CCTTGCTGCCATAAAGTCCC		58.89	55
TLR8 (exon)	forward	ATGCGGGAACATAAGAGATC	3994	55.01	45
TLR8 (exon)	reverse	GTGAACCATGACTTAACATCAG		55.12	40.91

Supplementary Figure 1: AlphaFold2 predictions of the shortened polypeptides of TLR6 of both leopard (aaPaPa-TLR6*47) and cheetah (aaAcJu-TLR6*02). For the leopard nucleotide allele aaPaPa-TLR6*47_01 was used and for the cheetah nucleotide allele aaAcJu-TLR6*02_01 was used as input. Confidence estimates of the predicted polypeptide structures (plDDT) range from <50 (very low confidence) to >90 (high confidence).

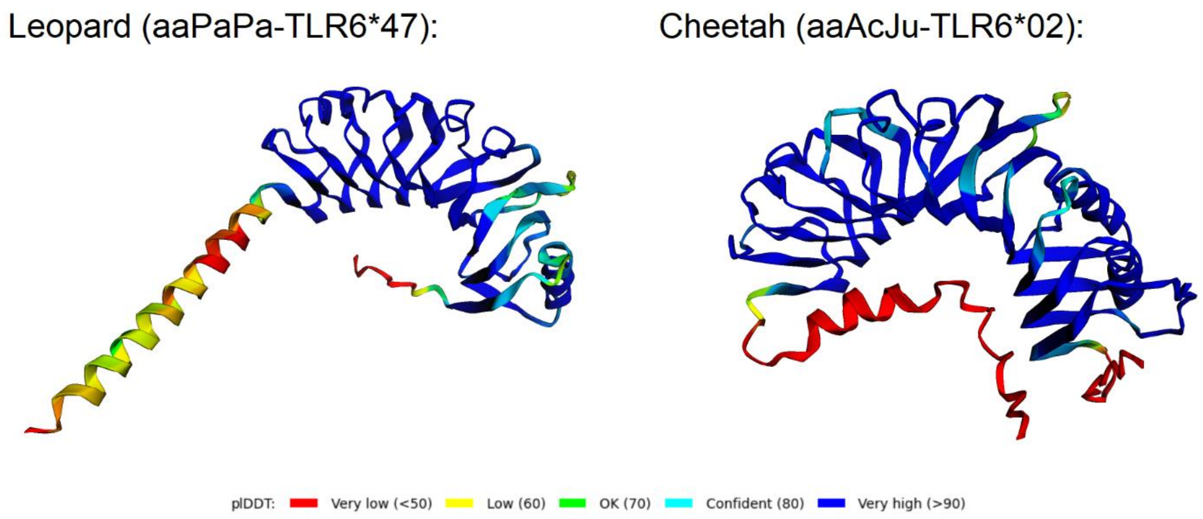

Supplementary Figure 2: Naming scheme of the nuclear alleles and resulting polypeptides.

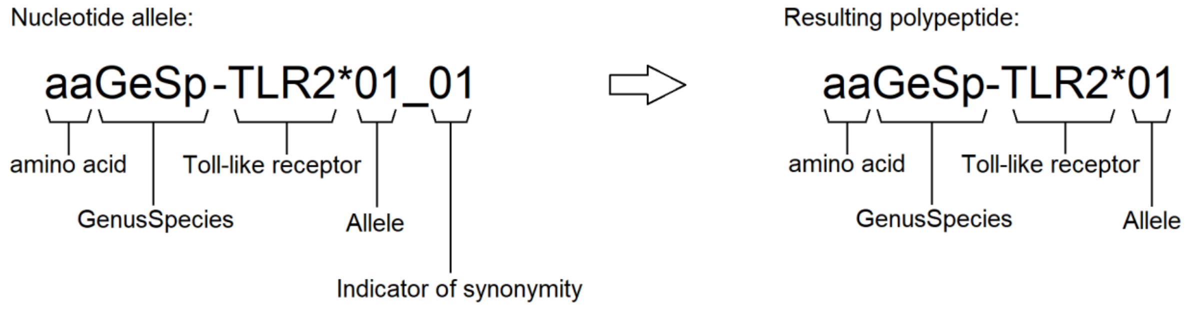

Script 1: Bash script used for read mapping and variant calling.

```
#!/bin/bash
#       Usage:
#               Mapping and variant calling
#       Needed:
#       TLR_reference.fasta NAME_1.fastq.gz NAME_2.fastq.gz
#
#
#
#
##################### MAPPING/VARIANT CALLING########################

cd /gpfs/data/fs71733/meissnerr/genomes/cheetah_genomes_ssp #working directory
picard CreateSequenceDictionary R=TLRs_consensus-+150.fasta O=TLRs_consensus-+150.dict #makes reference sequence dictionary for GATK
samtools faidx TLRs_consensus-+150.fasta #creates faidx indices
bwa index TLRs_consensus-+150.fasta #builds indices used for mapping with bwa
bowtie2-build TLRs_consensus-+150.fasta leopard_SR #creates dictionary for bowtie2

for file in *_1.fq.gz;do
	bn=`basename $file _1.fq.gz`

	#First Haplotype
	bwa mem -t 40 -T 20 -v 1 -L 5 -M TLRs_consensus-+150.fasta ${bn}_1.fq.gz ${bn}_2.fq.gz | samtools view -@ 40 -b - | samtools sort -n -@ 40 - | samtools fixmate -O bam - ${bn}.bam #mapping all reads to reference with soft clipping enabled and sort after name
	samtools flagstat ${bn}.bam #run basic stats
	samtools view -@ 40 -F 4 -b ${bn}.bam > ${bn}.sorted.no_unmapped.bam #removing all unmapped reads
	bedtools bamtofastq -i ${bn}.sorted.no_unmapped.bam -fq ${bn}.sorted.no_unmapped.bam.fastq #converts .bam back to .fastq for bowtie2 --end-to-end
	bowtie2 -q ${bn}.sorted.no_unmapped.bam.fastq --end-to-end -x leopard_SR -S ${bn}.bowtie2.no_unmapped.sam #maps all reads with soft clipping disabled
```

samtools view -u ${bn}.bowtie2.no_unmapped.sam | samtools sort -o ${bn}.bowtie2.sorted.no_unmapped.bam #uncompresses .sam, sorts abd converts to .bam

samtools index ${bn}.bowtie2.sorted.no_unmapped.bam #indexes .bam

picard AddOrReplaceReadGroups INPUT=${bn}.bowtie2.sorted.no_unmapped.bam OUTPUT=${bn}.with_readgroups.sorted.no_unmapped.bam SORT_ORDER=coordinate RGLB=L001 RGPL=Illumina RGPU=03500 RGSM=${bn} #adds read group information to reads

picard BuildBamIndex I=${bn}.with_readgroups.sorted.no_unmapped.bam #creates index for .bam

mv ${bn}.with_readgroups.sorted.no_unmapped.bai ${bn}.with_readgroups.sorted.no_unmapped.bam.bai #renames .bam to .bam.bai

java -jar /home/fs71733/meissnerr/anaconda3/envs/HaploCalling/opt/gatk-3.8/GenomeAnalysisTK.jar -T RealignerTargetCreator -R TLRs_consensus-+150.fasta -I ${bn}.with_readgroups.sorted.no_unmapped.bam -o ${bn}.intervals #marks wrongly aligned reads around an indel, saves information in .intervals

java -jar /home/fs71733/meissnerr/anaconda3/envs/HaploCalling/opt/gatk-3.8/GenomeAnalysisTK.jar -T IndelRealigner -R TLRs_consensus-+150.fasta -I ${bn}.with_readgroups.sorted.no_unmapped.bam -targetIntervals ${bn}.intervals -o ${bn}.indel.with_readgroups.sorted.no_unmapped.bam #realigns reads around indel based on .intervals

samtools indexstats

samtools index ${bn}.indel.with_readgroups.sorted.no_unmapped.bam #indexes .bam

picard MarkDuplicates INPUT=${bn}.indel.with_readgroups.sorted.no_unmapped.bam OUTPUT=${bn}.dedup.indel.with_readgroups.sorted.no_unmapped.bam METRICS_FILE=${bn}.dedupStats.txt OPTICAL_DUPLICATE_PIXEL_DISTANCE=100 REMOVE_DUPLICATES=true #removes read duplicates optical

samtools index ${bn}.dedup.indel.with_readgroups.sorted.no_unmapped.bam #indexes .bam

bcftools mpileup -Q 20 -A -f TLRs_consensus-+150.fasta ${bn}.dedup.indel.with_readgroups.sorted.no_unmapped.bam | bcftools call -mv > ${bn}_1.vcf #produces textual format from alignment, calls and stores SNP/indel data in .vcf

bcftools filter -s LowQual -e '%QUAL<20 || DP>10' ${bn}_1.vcf > ${bn}_2.vcf #filters -vcf and removes low quality called SNPs/indels

bgzip ${bn}_2.vcf

tabix -p vcf ${bn}_2.vcf.gz #indexes .vcf for TAB-delimited genome position files

bcftools consensus -f TLRs_consensus-+150.fasta ${bn}_2.vcf.gz > ${bn}_consensus1.fasta #creates first haplotype consensus

#Second Haplotype

picard CreateSequenceDictionary R=${bn}_consensus1.fasta O=${bn}_consensus1.dict #makes reference sequence dictionary for GATK for second haplotype based on first haplotype consensus

samtools faidx ${bn}_consensus1.fasta #creates faidx indices

bowtie2-build ${bn}_consensus1.fasta leopard_SR_first_haplotype #creates dictionary for bowtie2

bowtie2 -q ${bn}.sorted.no_unmapped.bam.fastq --end-to-end -x leopard_SR_first_haplotype | samtools sort -@ 40 -O BAM -o ${bn}.sorted.no_unmapped_2.bam - #maps all reads with soft clipping disabled to new reference and sorts them

samtools index ${bn}.sorted.no_unmapped_2.bam #indexes .bam

picard AddOrReplaceReadGroups INPUT=${bn}.sorted.no_unmapped_2.bam OUTPUT=${bn}.with_readgroups.sorted.no_unmapped_2.bam SORT_ORDER=coordinate RGLB=L001 RGPL=Illumina RGPU=03500 RGSM=${bn} ##adds read group information to reads

picard BuildBamIndex I=${bn}.with_readgroups.sorted.no_unmapped_2.bam #creates index for .bam

mv ${bn}.with_readgroups.sorted.no_unmapped_2.bai ${bn}.with_readgroups.sorted.no_unmapped_2.bam.bai ##renames .bam to .bam.bai

java -jar /home/fs71733/meissnerr/anaconda3/envs/HaploCalling/opt/gatk-3.8/GenomeAnalysisTK.jar -T RealignerTargetCreator -R ${bn}_consensus1.fasta -I ${bn}.with_readgroups.sorted.no_unmapped_2.bam -o ${bn}.intervals #marks wrongly aligned reads around an indel, saves information in .intervals

java -jar /home/fs71733/meissnerr/anaconda3/envs/HaploCalling/opt/gatk-3.8/GenomeAnalysisTK.jar -T IndelRealigner -R ${bn}_consensus1.fasta -I ${bn}.with_readgroups.sorted.no_unmapped_2.bam -targetIntervals ${bn}.intervals -o ${bn}.indel.with_readgroups.sorted.no_unmapped_2.bam #realigns reads around indel based on .intervals

samtools idxstats

samtools index ${bn}.with_readgroups.sorted.no_unmapped_2.bam #indexes .bam

samtools index ${bn}.indel.with_readgroups.sorted.no_unmapped_2.bam ##indexes .bam

picard MarkDuplicates INPUT=${bn}.indel.with_readgroups.sorted.no_unmapped_2.bam OUTPUT=${bn}.dedup.indel.with_readgroups.sorted.no_unmapped_2.bam METRICS_FILE=${bn}.dedupStats.txt OPTICAL_DUPLICATE_PIXEL_DISTANCE=100 REMOVE_DUPLICATES=true #removes read duplicates optical

samtools index ${bn}.dedup.indel.with_readgroups.sorted.no_unmapped_2.bam #indexes .bam

bcftools mpileup -Q 20 -A -f ${bn}_consensus1.fasta ${bn}.dedup.indel.with_readgroups.sorted.no_unmapped_2.bam | bcftools call -mv > ${bn}_1_2.vcf #produces textual format from alignment, calls and stores SNP/indel data in .vcf

```
bcftools filter -s LowQual -e '%QUAL<20 || DP>10' ${bn}_1_2.vcf > ${bn}_2_2.vcf #filters -vcf and removes low quality called SNPs/indels

bgzip ${bn}_2_2.vcf

tabix -p vcf ${bn}_2_2.vcf.gz #indexes .vcf for TAB-delimited genome position files

bcftools consensus -f ${bn}_consensus1.fasta ${bn}_2_2.vcf.gz > ${bn}_consensus2.fasta #creates second haplotype consensus


done


for file in *_consensus1.fasta; do

name=$(echo "$file" | cut -d "_" -f 1) ;

sed --in-place "s/leopard/$name/g" $file && sed --in-place 's/allele/allele_1/g' $file
```
